# How the brain segments experience: ERP evidence of event boundaries enhancing memory formation in narratives

**DOI:** 10.3758/s13415-026-01399-0

**Published:** 2026-03-16

**Authors:** Doruntinë Zogaj, Regine Bader, Axel Mecklinger

**Affiliations:** Experimental Neuropsychology Unit, Department of Psychology, Saarland University, Campus A2.4, 66123 Saarbrücken, Germany

**Keywords:** Event boundaries, Episodic memory, EEG, Prediction error, Subsequent memory effect

## Abstract

Event boundaries are known to have an impact on how discrete events are remembered; however, the neural mechanisms supporting memory for boundaries themselves remain poorly understood. This study investigated how both event boundaries and preceding information are processed and remembered while listening to naturalistically spoken narratives. We recorded participants’ neural responses (event-related potentials) while they listened to stories where a critical word signaled either a predictable (no-event boundary) or an unpredictable (event boundary) action. Critical words in the boundary condition were better remembered than those in the no-event boundary condition and elicited a larger N400 amplitude. Crucially, a subsequent memory effect was observed only in the boundary condition, with remembered critical words eliciting more negative N400s than forgotten ones, highlighting the role of increased demands in conceptual semantic processing in episodic memory encoding. Furthermore, a retrograde subsequent memory effect emerged also exclusively in the boundary condition, with more negative amplitudes to critical words when preceding information was later remembered, consistent with the notion that boundaries trigger rapid reinstatement of a recently experienced event. These findings provide compelling evidence that event boundaries act as “cognitive anchor points” that enhance the encoding of new information and also contribute to the strengthening of recently encoded events.

## Introduction

Although the world is experienced as a continuous stream of information, individuals naturally organize their memories into distinct and cohesive events. Research shows that people inherently segment ongoing experiences at points of contextual change (such as shifts in location, time, characters, causality, and goals) or prediction failure, so called event boundaries (Kurby & Zacks, 2008; Richmond & Zacks, 2017). According to event segmentation theory (EST), event boundaries arise when the active predictive model becomes inaccurate, triggering a prediction error that prompts a transient shift in attention to update the model and encode the new event (Reynolds et al., 2007; Zacks et al., 2009, 2011). This account also suggests that the increase in externally oriented attention at event boundaries enhances memory for information encountered during these contextual shifts. Supporting this view, there is evidence that event boundaries improve memory, especially for information encountered near a boundary (Newtson & Engquist, 1976; Swallow et al., 2009). These enhancements extend beyond item memory to associative memory processes (Clewett et al., 2020; Heusser et al., 2018) consistent with the idea that boundaries broaden attention and promote the integration of contextual information into memory (DuBrow et al., 2024).

While EST proposes that event boundaries are primarily triggered by a prediction error (Zacks et al., 2007), the universality of prediction error as the sole trigger for event boundary detection is a subject of ongoing debate. For instance, Pettijohn and Radvansky (2016) showed that situation model updating occurs even when narrative shifts are foreshadowed. Yet, readers still responded more slowly to probes about preshift events, suggesting that updating at boundaries can occur without a strong prediction error. Furthermore, the contextual stability account suggests that segmentation is primarily driven by transitions across stable contexts (Clewett et al., 2019; DuBrow & Davachi, 2016). Indeed, recent work reported that segmentation remains robust for both predicted and unpredicted transitions, a result interpreted as supporting the pivotal role of contextual stability in event segmentation (Güler et al., (2025). A relevant, alternative perspective on how individuals construct mental representations in an unfolding narrative is provided by the Structure Building Framework (SBF) (Gernsbacher, 1990). The SBF proposes that readers build coherent mental structures via three mechanisms: initially laying the foundation of a new mental substructure, subsequently mapping coherent incoming information onto this active structure, and finally initiating a shift to construct a new structure when mapping fails due to inconsistency with the current context or irrelevance (Gernsbacher, 1990; Zacks et al., 2009).

Although event boundaries can arise from multiple types of contextual shifts, the present study focused specifically on unpredictable event boundaries, operationalized as a change in the action verb that signaled an abrupt shift in the character's intentionality or goal, a key dimension monitored during situation model updating (Pettijohn & Radvansky, 2016).

Neuroimaging studies using naturalistic videos show that regions such as the hippocampus and specific neocortical areas exhibit increased activity at boundaries, reflecting their role in processing perceived event changes (DuBrow & Davachi, 2016). This boundary-related activation appears to trigger a cascade of memory-related processes, as evidenced by increased medial temporal lobe activation surrounding event transitions (Ben-Yakov et al., 2018). A key component of this cascade is the hippocampal activation at the boundaries, which has been reported to reflect the rapid memory replay of the entire just-experienced event (Ben-Yakov & Dudai, 2011; Griffiths & Fuentemilla, 2020). Further evidence for the role of boundary-evoked neural activity comes from event-related potential (ERP) studies. Silva et al. (2019) found that during movie viewing more negative left-lateralized ERP responses elicited at event boundaries were specifically associated with the recall of information preceding the boundary. They proposed that this boundary-locked ERP activity reflects the successful detection of a context shift between the current and just-encoded event, which triggers rapid memory reactivation of the preceding event. However, the latter study does not address which processes contribute to memory formation for the boundary element itself. Another study demonstrated that detecting contextual shifts triggers rapid memory reinstatement of just-encoded information, supporting the binding of preceding events into a long-term memory representation of continuous experience (Sols et al., 2017). Similarly, Wu et al. (2022) reported that rapid memory reinstatement at the offset of a meaningful episodic sequence predicted later memory recall for that specific episode. These findings suggest that rapid, online memory reinstatement may be a critical neural mechanism supporting the long-term retention of just-encoded events. It should be noted, however, that these studies used different methodological approaches to assess memory reactivation at event boundaries than the one adopted in the present experiment.

Using ERPs, the N400 serves as a critical marker in understanding how the brain processes event boundaries. The N400 is a negative-going ERP component peaking approximately 400 ms poststimulus onset, commonly displaying a centroparietal scalp distribution (Federmeier & Kutas, 1999; George et al., 1997). Its amplitude is functionally recognized to be modulated by the extent to which a word is expected within a sentence or broader context (Kutas & Federmeier, 2011) and reflects the ease of accessing and retrieving conceptual knowledge or the integration of an event into its preceding semantic or discourse context (Kutas & Federmeier, 2000; Lau et al., 2008). Delogu et al. (2018) observed larger N400 amplitudes for target words marking “coarse event boundaries" (less predictable actions initiating a goal shift) compared with "fine event boundaries" (more predictable actions continuing current goal). The N400 effect reflected the increased demands on the activation of conceptual knowledge at boundaries, where incoming information was unpredictable or semantically incongruent with the ongoing mental model. However, it remains unclear how exactly the activation of conceptual knowledge contributes to the successful encoding not only of preceding information but also of the boundary information itself. To address this gap, the present study examines how event boundaries interact with memory formation processes and explores their neural underpinnings. Our work is the first to investigate how both event boundary information and the preceding context are processed and remembered within a single experiment by using an ERP subsequent memory approach. With this approach, we utilize the high temporal resolution of ERPs and compare neural activity recorded during story encoding for words that were later remembered versus those that were later forgotten in a subsequent recognition memory test (Cohn et al., 2014; Mecklinger & Kamp, 2023).

During the study phase, participants listened to short stories each consisting of five sentences describing a common activity (e.g., going to the library), with the third sentence containing a critical word indicating either a predictable action (e.g., reading) marking no-event boundary or unpredictable action (e.g., shopping) marking event boundary, followed by two confirming sentences. As the critical word indicated a change in action, e.g., “shopping” after “borrowing books,” the boundary clearly signifies a shift in the protagonist's goal denoting a new event. Such an unpredictable change in action verb is thought to function as a prediction error, signaling a discontinuity with the ongoing event model and prompting its update. Memory was tested using an old/new recognition task for two words preceding the critical word, the critical word itself, and new words. Here, we aimed to combine ecologically valid auditory narrative text stimuli including clearly defined event boundaries, with an ERP subsequent memory effect (SME) approach to investigate how event boundaries shape the online formation of event memories. Event-related potential SMEs are typically identified as positive-going components, meaning subsequently remembered items elicit more positive amplitudes than forgotten items, and they temporally dissociate various encoding mechanisms (Mecklinger & Kamp, 2023). The current study focuses on two specific SME components defined by their spatiotemporal characteristics: the early parietal SME typically emerges between 350 and 500 ms as a positive deflection over parietal sites. It is functionally interpreted as reflecting the effective integration of multiple features into a rich item memory representation (Mecklinger & Kamp, 2023). This early parietal effect is often observed when encoding benefits from semantic knowledge or coherent context (Bridger & Wilding, 2010; Otten et al., 2007). The late frontal SME is a prolonged frontal positivity typically observed from around 550 ms onward, functionally associated with elaborative encoding strategies that emphasize relational processing of multiple study items (Bauch & Otten, 2012).

Building on evidence of boundary-related memory enhancements, we expected better memory for critical words in the boundary condition than in the no-boundary condition. Coherent contexts, in addition, have been shown to provide rich associative links that enable strong predictions about upcoming information, thereby easing the processing of expected words and enhancing the encoding of congruent events (Craik & Lockhart, 1972; Greve et al., 2019; Höltje & Mecklinger, 2022). Thus, if coherent contexts enhance the encoding of expected information, no-boundary critical words should exhibit a parietal subsequent memory effect (SME) (Höltje & Mecklinger, 2022). In contrast, boundary words, which are unexpected and contextually incongruent, were expected to elicit an enlarged N400 and a late frontal SME, reflecting semantic prediction violation and integration effort, respectively (Delogu et al., 2018; Höltje & Mecklinger, 2022).

Beyond examining how memory for event boundaries is enhanced in ecologically valid contexts, such as naturalistic spoken narratives, this study also investigated how boundary processing influences the retention of preceding words. Consistent with the idea that boundaries function as brief breakpoints, preceding information may be rapidly replayed at event boundaries, thereby facilitating the binding of preceding events in memory (Bilkey & Jensen, 2021; Clewett & Davachi, 2017). Previous ERP research (Silva et al., 2019) suggests that boundaries support the consolidation and integration of preceding events into long-term memory. We therefore hypothesized that in the boundary condition, participants would be more likely to remember one preceding word if the other was also remembered (conditionalized item memory) compared with the no-boundary condition. Additionally, we expected that the ERP response to the critical item would show greater positivity when both preceding words were remembered, compared with when only one or none were remembered, reflecting retrograde successful memory encoding. This effect was expected to be present in the boundary condition but not in the no-boundary condition.

## Method

### Pre-registration

This study was preregistered with AsPredicted, the preregistration details can be accessed at https://aspredicted.org/9mzn-wz5h.pdf.

### Participants

A sample (*N* = 45) of young adults was recruited from the student population of Saarland University via flyers and local databases. For current analysis, data from five participants were excluded due to the following reasons: low performance on comprehension questions (*n* = 1; see *Procedure* section for details), insufficient free-artifact EEG trials (*n* = 3), and technical issues (*n* = 1). As a result, the final sample consisted of 40 German native speakers (31 females, 6 males, and 3 other, *M* = 22.5 years, *SD* = 2.53, *range* = 18–28). All participants had normal or corrected-to-normal vision and hearing, were right-handed as assessed by the Edinburgh Handedness Inventory (laterality quotient ≥ 50, Oldfield, 1971) and reported no neurological and/or psychiatric conditions. Participants provided informed consent and were compensated with €12 per hour or course credit. Participants were debriefed after the experiment.

The sample size was determined through a power analysis using R package pwr (Champely et al., 2020) for a paired *t*-test on the effect of interest, i.e., the SME effect, based on Höltje and Mecklinger (2022), (*dz* = 0.49, α = 0.05, 1-β = 0.80). This analysis indicated a required sample size of 37.06, which resulted in a final sample size of 40 participants for counterbalancing purposes. Overall, exclusion criteria included item memory performance and comprehension question accuracy at chance level, as determined by a binomial test. Additionally, ERP datasets with fewer than seven artifact-free trials per condition were excluded for the SME of critical words, and fewer than five artifact-free trials^1^ were excluded for the retrograde SME. The experimental procedures were approved by the ethics board of the Faculty of Human and Business Sciences at Saarland University (nr: 21–28).

### Material

**Fig. 1:**
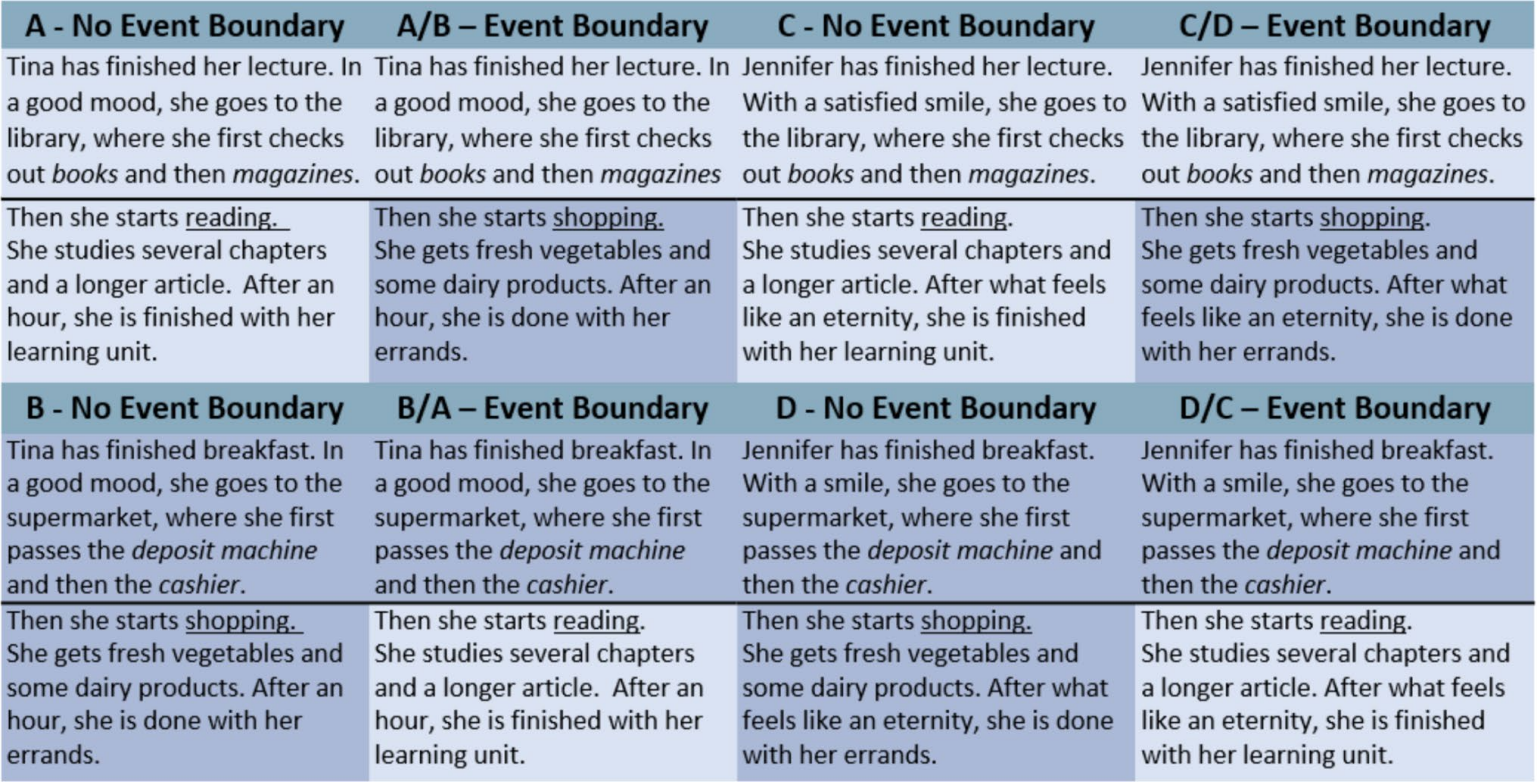
A sample of story pairs, translated from German to English. The figure provides an example of the stimulus material used in the study. Critical words are underlined. The preceding words which also appeared later in the memory test are in italics. This design also ensured that participants were not exposed to the same test words more than once

Initially, we created 142 stories, adapted from Delogu et al. (2018). The first step in creating the stimulus material was to construct two no-event boundary stories, each describing a different activity (e.g., reading vs. shopping), as seen in Fig. 1. Story A and Story B were designed to share a similar introductory structure and narrative pacing, with parallel sentence structures and matched timing of key words, allowing for a recombination of their halves. Event boundary stories were created by combining the first part of Story A with the second part of Story B and vice versa, thereby introducing an event boundary by disrupting the natural sequence of activities. Each story consisted of five sentences. The first sentence provided a short introduction, setting up the context for the upcoming activity. The second sentence introduced a typical location (e.g., library or supermarket) and described a sequence of actions related to the activity. This sentence contained two specific words that were later tested in the memory task as the “preceding words.” The third sentence always began with “Then she/he starts” (German: “Dann beginnt sie mit dem.…”) acting as a cue for the upcoming event. At the end of this sentence, a critical word was introduced, referring either to a predictable action (e.g., reading after borrowing books), marking no-event boundary, or to a less predictable action (e.g., shopping after borrowing books), marking an event boundary. Note that in both no-boundary and boundary conditions, the critical word consistently followed the article “dem,” a content-free function word, ensuring that the baseline preceding the critical word was balanced across conditions. The fourth and fifth sentences reinforced the activity mentioned in the third sentence. To ensure a sufficient number of stories per participant and condition, while avoiding repetition of the words later used in the memory test, two additional versions (C and D) were created. These stories maintained the same activities but included slight modifications, such as different character names and minor wording changes (e.g., “Mood” to “Smile” and “Hour” to “Eternity”). As with the previous stories, event boundary stories were constructed by combining the first part of Story C with the second part of Story D, and vice versa. This resulted in a total of 568 stories.

To select the final stimulus material for the EEG study, two rating studies were conducted with independent samples of participants recruited via Prolific Academic Ltd., using lab.js (Henninger et al., 2022) and hosted on Open Lab (Shevchenko, 2022). Each participant took part in only one of the rating studies and was paid £8.00 for the first rating test or £10.00 for the second rating test. The stimulus material was divided into four lists, with participants being presented with only one story from each version (e.g., A and D), ensuring that no preceding and critical word appeared more than once.

The aim of the first rating study was to ensure that the critical words in the no-event boundary condition were perceived as a natural continuation of the context, and the critical words in the event boundary condition were seen as unexpected or incongruent with the context. Each group was presented with the first three sentences of either a no-event boundary or an event boundary story. Four groups of participants (one group with 18 participants, one with 17, and two with 15) rated the stories on a scale from 1 (very poorly) to 4 (very well) in response to the question, “How well does the last sentence fit into the previous context?” To further assess the overall coherence of the stories, a second rating study was conducted with four new groups, each consisting of 15 participants. In this study, participants were presented with the entire story and asked to rate it on a scale from 1 (very poorly) to 4 (very well) in response to the question, “To what extent is the story coherent?” From the 568 initial stories, we selected 480 that were rated coherent for no-event boundaries and incoherent for event boundaries in both tests. The no-event boundary stories were rated as coherent (*M* = 3.5, *SD* = 0.7), while the event boundary condition stories were rated as less coherent (*M* = 1.6, *SD* = 0.8). The four final stimulus lists, each containing 120 stories (both no-event boundary and event boundary), were mixed with 22 filler stories that had been rated as neither coherent nor incoherent in the rating tests. Filler stories were included to serve as the basis for comprehension questions.

All stimuli of the study phase were presented auditorily through loud speakers at a fixed volume of 40 dB for all participants, while stimuli in the test phase were presented visually in black Calibri font at 18-point size against a white background. Each story was recorded via Audacity (R) version 3.5.1 by a female native German speaker who read the stories as natural as possible, with her normal speed of speaking. The stories had an average duration of 17 s, while the critical words lasted on average 622.75 ms (*SD* = 137.05 ms), ranging from 328.52 ms to 1,028.47 ms. Auditory files were edited by using Praat (Boersma & Weenink, 2023) to ensure consistent sentence-to-sentence intervals across all stories. Additionally, a 300 ms pause was inserted between the critical word and the start of the fourth sentence in every story to maintain a brief period of silence in both conditions. It is important to note that across the four lists, the duration of critical words did not significantly differ between conditions. For Lists A and C, no-boundary critical words had a mean duration of 619 ms (*SD* = 126 ms) and boundary critical words 627 ms (*SD* = 149 ms). In Lists B and D, these values were assigned in the opposite way due to counterbalancing. During the test phase, participants were shown 120 critical words, 240 preceding words and 360 new, previously not presented words that were matched in terms of length and lexical frequency with presented words.

### Procedure

**Fig. 2:**
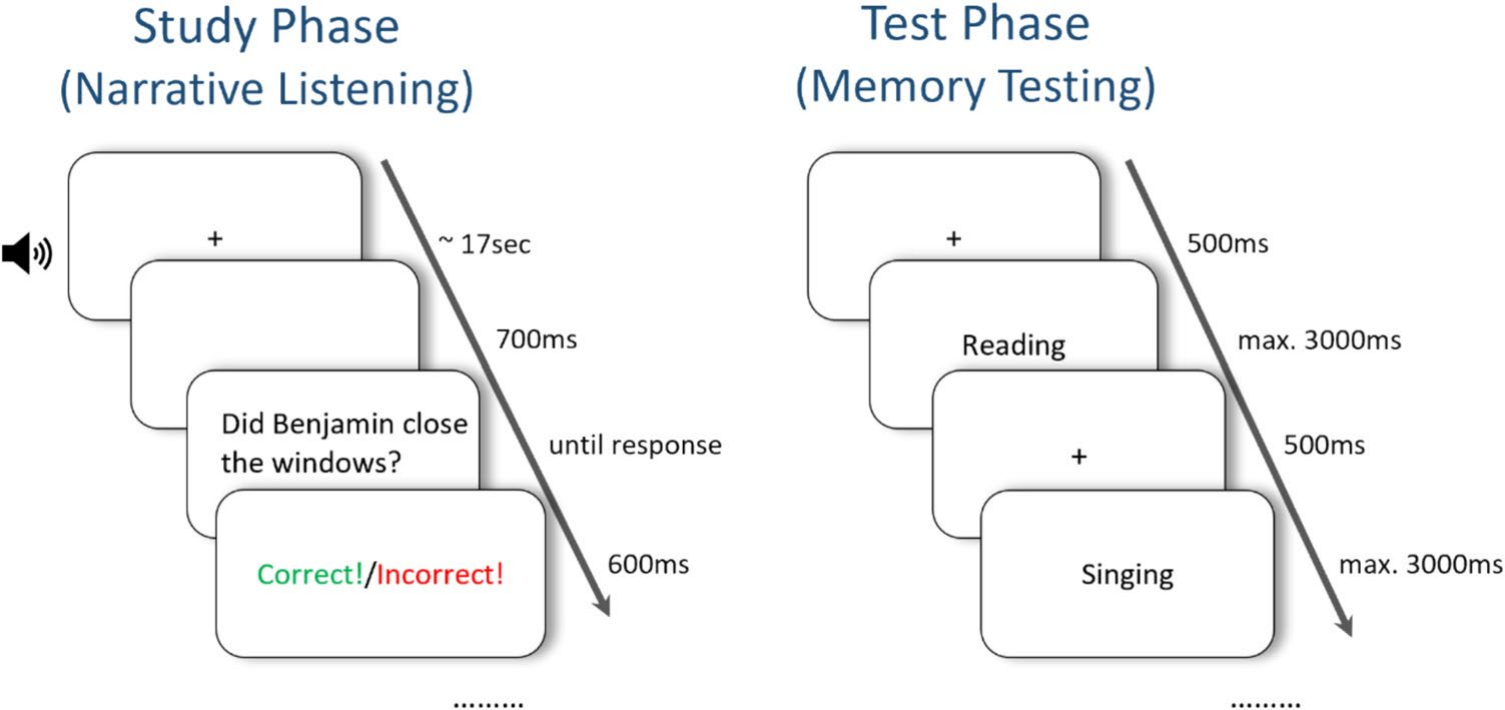
Trial procedures of the study and test phases. The figure depicts the trial procedures of the learning phase and the recognition memory test. The English translations of the stimulus materials are provided for illustrative purposes only, as all stimuli were presented in German

After assessing demographical variables and the inclusion criteria, electroencephalography (EEG) was applied. During the experiment, participants sat in front of a 24-inch monitor with a resolution of 1920 × 1080 px at a distance of approximately 80 cm, inside a sound-absorbing and electrically shielded chamber. The experiment was programmed and conducted using E-Prime 2.0 (Psychology Software Tools, Pittsburgh, PA). The experimental session lasted approximately 1.5 h and contained eight blocks, each consisting of a study phase (17–18 stories) followed by a test phase (90 words). The trial procedures of the study and test phase are illustrated in Fig. 2.

The experiment began with a short practice session consisting of four study trials and 12 test trials to familiarize participants with the task. During each study trial, while the stories were played out loud, the participants were instructed to look at the fixation cross on the screen and to listen to the stories for comprehension and avoid blinking. A 700 ms blank screen followed the audio file. In 15.49% of the trials, a yes/no comprehension question appeared (e.g., Did Benjamin close the windows?) and was terminated when the participants responded by pressing one of two buttons (“c” or “m”), counterbalanced across subjects. Each response was followed by feedback (“correct” or “incorrect”) for 600 ms. The inclusion of comprehension questions served to maintain participants’ attention and to verify that they followed the story content attentively. In the test phase, participants were asked to indicate which words were from the sentences they heard previously, using an old/new recognition memory task. An old word means that it appeared in one of the stories in the immediately preceding study phase, whereas new words did not appear in any of the stories. The task was to categorize them as definitely old, probably old, probably new, or definitely new by either pressing keys 1 through 4 or 4 through 1, alternating between subjects. A trial started with a 500 ms fixation cross, followed by a test word, which appeared up to 3,000 ms but was terminated as soon as the participants responded. Stimulus presentation order was randomized for study and test phases.

### Electrophysiological recording & preprocessing

The EEG was recorded from 28 Ag/AgCl scalp electrodes (Fp1, Fp2, F7, F3, Fz, F4, F8, FC5, FC3, FCz, FC4, FC6, T7, C3, Cz, C4, T8, CP3, CPz, CP4, P7, P3, Pz, P4, P8, O1, O2, and A2) mounted in an elastic cap (Easycap) and placed according to the 10–20 system. Data were recorded using AFz as a ground electrode and the left mastoid electrode (A1) as a reference electrode via a 16bit BrainAmp amplifier (Brain Products). Electrooculogram (EOG) was recorded using four additional electrodes positioned above and below the right eye, as well as at the outer canthi of both eyes. The signals were band-pass filtered between 0.016 and 100 Hz and digitized at a sampling rate of 500 Hz. All electrode impedances were kept below 5 kΩ. Offline, the data were processed using Brain Vision Analyzer 2.2 (Brain Products). As a first step, the data were visually inspected manually to identify and remove excessive (i.e., muscular) artifacts from the raw data. Next, data were filtered by using a 0.1–30 Hz butterworth-filter (order: 4) and a notch-filter (50 Hz). An independent component analysis (ICA) using the classic biased restricted info-max algorithm implemented in Brain Vision Analyzer 2.2 (Brain Products) was employed to identify and correct the EOG and cardiac artifacts. Then, the signal was re-referenced to the average of both mastoid electrodes and segmented into epochs from − 200 to 1,000 ms around test word onset, including a 200 ms baseline. Following baseline correction, a semi-automatic artifact rejection was applied. Segments containing absolute amplitudes larger/smaller than ± 70 µV, with a maximal voltage difference of 100 µV within a 200 ms interval, or with a voltage step larger than 30 µV/ms were rejected. Lastly, any remaining segments that contained excessive alpha-activity were manually excluded. Finally, we created participant-wise averages for each condition (critical words and preceding words: no-event boundary, event boundary) and computed grand-average waveforms. After exporting the low-pass filtered (12 Hz) grand averages, for plotting the ERP waveforms we used “ggplot2” from the “tidyverse” package in R.

To calculate average N400 effects, *M* = 55.98 trials (*SD* = 5.53, range 39–64) were used in the no-event boundary condition and *M* = 56.27 trials (*SD* = 5.43, range 46–64) in the event boundary condition. The average ERPs for the SME analyses were calculated for subsequent hits (old words identified as old) and subsequent misses (old words identified as new) for each condition (no-event boundary and event boundary condition), respectively. The ERPs elicited by subsequent hits were based on *M* = 35.725 trials (*SD* = 9.23, range 20–55) in the no-event boundary condition and on *M* = 37.125 trials (*SD* = 7.42, range 23–50) in the event boundary condition. For ERPs of subsequent misses, *M* = 20.175 trials (*SD* = 7.98, range 7–40) were used in the no-event boundary condition and *M* = 19.15 trials (*SD* = 7.25, range 7–37) were used in the event boundary condition. As preregistered, we planned to define factor levels for the retrograde SME analyses based on memory performance and trial counts (i.e., 0, 1, or 2 preceding words remembered, or alternatively comparing 0 vs. 2 or 0 vs. 1). On average, trial counts across the three levels were similar; however, to enable a stronger contrast between the two extreme levels, we opted to compare trials in which both preceding words were remembered with those in which none were remembered. For the retrograde SME effects, the ERPs at critical words when both preceding words were remembered were based on *M* = 19.6 trials (*SD* = 8.89, range 8–39) in the no-event boundary condition and on *M* = 19.37 trials (*SD* = 6.36, range 9–36) in the event boundary condition. For ERPs of none preceding words remembered, the corresponding trial numbers were *M* = 16.14 trials (*SD* = 6.95, range 5–32) in the no-event boundary condition and *M* = 16.14 trials (*SD* = 6.37, range 5–34) in the event boundary condition.

### Statistical analysis

All analyses were conducted using R (version 4.4.1; R Core Team, 2024) and RStudio (version 2024.04.0; RStudio Team, 2024). The packages “tidyverse” (Wickham et al., 2019), “reshape2” (Wickham, 2007), dplyr (Wickham et al., 2021), lme4 (Bates et al., 2015), and afex (Singmann et al., 2024) were used. All analyses followed those stated in the pre-registration; deviations from the pre-registration are marked accordingly. The significance criterion was set to *p* <.05. Only significant effects involving the factors event boundary and/or memory are reported.^2^

Item memory performance was assessed using hit rates and false-alarm (FA) rates. The hit rate was calculated as the proportion of previously heard old words correctly identified as old, and the FA rate was the proportion of new words incorrectly classified as old. Responses on the confidence scale were collapsed into “old” (“definitely old” and “maybe old”) and “new” (“definitely new” and “maybe new”). Because new words do not belong to any condition, hit rate served as the condition-specific performance measure, whereas the Pr scores (hits—FA) was used as the main index of item memory performance in statistical analysis. The Pr scores in the item memory task were compared by using paired *t*-tests.

Responses made before or at 200 ms were excluded from all behavioral analyses. To further analyze the potential interaction between event boundary (boundary vs. no-boundary) and confidence (high vs. low) on memory performance, we conducted a two-way ANOVA. Conditionalized item memory was evaluated by determining the probability of remembering the first word preceding the critical word, given that the second preceding word was remembered, and vice versa. We used generalized linear mixed-effects models (GLMMs) with binomial distribution as implemented in the lme4 package in R to assess whether the likelihood of remembering one word was influenced by memory for the other and whether this relationship was moderated by the presence of an event boundary (boundary vs. no boundary). The fixed effects included the event boundary, the accuracy of the other word, as well as their interactions. To control for potential linear trends across trials, centered word position was added as a predictor. Random effects included intercepts and, where possible, slopes for participants and for test words nested within block lists, to account for the hierarchical structure of the data. In case of nonconverging models or singularity issues, each model was simplified progressively using the least variance approach (Barr, 2013). *P*-values (significance criterion: *p* <.05) were determined using Wald test (via summary (glmer_model)).

For EEG data analysis a three-step procedure was used. First, as a manipulation check, we aimed to evaluate the boundary manipulation by examining the N400 effect, comparing ERPs between no-event boundary and event boundary trials. Second, we investigated whether critical words exhibited similar parietal and late frontal subsequent memory effects as reported in previous studies (Höltje & Mecklinger, 2022; Kamp et al., 2017). Finally, in the third step, we examined the retrograde subsequent memory effect for critical words, comparing trials where both preceding words were remembered with trials in which neither word was remembered.

Due to the temporal variability of ERPs in response to spoken language, we applied the collapsed localizers technique (Luck & Gaspelin, 2017) to determine the timing and scalp distribution of effects. To define the analysis time window for the N400, the ERP waveforms were first averaged across the two event boundary conditions, and the most negative peak within the 250–600 ms interval was identified at 486 ms over the posterior cluster. For the present experiment, the N400 was analyzed in the 411–561 ms interval, obtained by ± 75 ms around the peak (486 ms). In the following sections, for clarity and consistency, this interval (411–561 ms), will be referred to as the N400 time window. Following this window, two additional consecutive 200 ms time windows were explored.

The parietal and frontal subsequent memory effects (SME) were analyzed separately within all three-time windows, using event boundary, anterior–posterior distribution and memory as factors. To assess the retrograde SME, we applied the same three-time windows approach. All the analyses were conducted by using the anterior (Fp1, Fp2, F3, Fz, F4) and posterior (CP3, CPz, CP4, P3, Pz, P4) electrode clusters.

Within each time window, electrophysiological data were analyzed by using repeated-measures ANOVA and paired-samples t-tests. The Greenhouse–Geisser correction was applied to all repeated measures with more than one degree of freedom in the numerator, and in such cases, the corrected *p*-value is reported. Significant interactions were further examined by using lower-level ANOVAs and paired-samples *t*-tests. Effect sizes are reported as partial eta squared (*η*_*p*_^2^) for ANOVA results and Cohen’s *dz* for paired-samples *t*-tests.

## Results

### Behavioral results

On average 85.96% of comprehension questions were correctly answered, implying that the participants were engaged and actively listened to the stories.

**Fig. 3:**
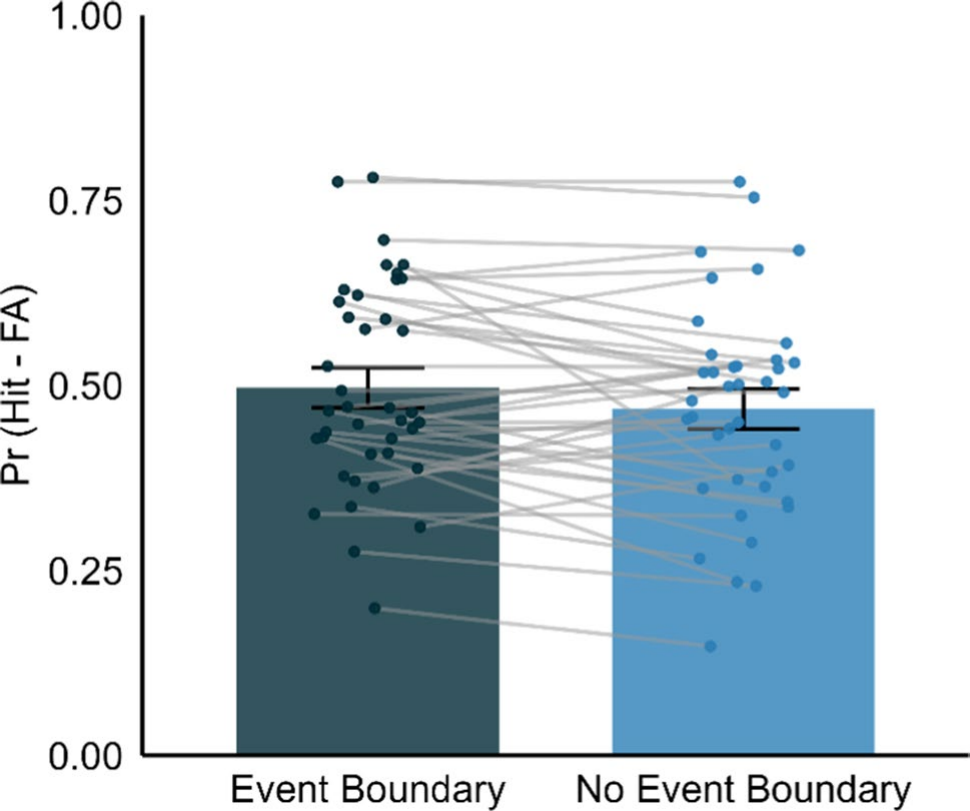
Pr scores for item memory. *Note*: Error bars represent 95% confidence intervals based on the standard error of the paired difference between conditions. Dots represent values for individual participants

Consistent with our hypothesis, item memory performance (Pr score) was significantly higher for critical words in the boundary condition compared to the no-boundary condition,* t*(39) = 2.17, *p* = 0.036, Cohen’s *d* = 0.34 (Table 1), indicating that the critical words were better remembered in the event boundary than in the no-event boundary condition. Mean item memory performance is illustrated in Fig. 3.

**Table 1:** Behavioral data—Item Memory

	All responses	
	Event boundary	No-event boundary
Pr score	0.50 (0.14)	0.47 (0.14)
Hit rate	0.67 (0.12)	0.64 (0.14)
FA rate	0.17 (0.10)	0.17 (0.10)

*Note*. Mean proportions of hits, false alarms, and *Pr* scores for each condition. Standard deviations are given in parentheses. *FA* = false alarm

To further analyze the potential relationship between confidence ratings, event boundary and memory performance, we conducted a two-way ANOVA with event boundary (boundary vs. no-boundary) and confidence (high vs. low) as factors. The analysis revealed a significant main effect of confidence (*F*(1, 39) = 24.83, *p* <.001, *η*_*p*_^2^ = 0.39), a main effect of boundary (*F*(1, 39) = 5.02, *p* =.03, *η*_*p*_^2^ = 0.11), and a significant interaction between event boundary and confidence (*F*(1, 39) = 9.14, *p* =.004, *η*_*p*_^2^ = 0.19). To explore this interaction, we examined the effect of event boundaries on memory separately for high-confidence and low-confidence responses. A *t*-test revealed that for low-confidence responses, memory did not differ between boundary and no-boundary trials, *t*(39) = − 1.03, *p* =.3, *d* = − 0.11. For high-confidence responses, memory performance was better for boundary, than for no-boundary trials, *t*(39) = 4.5, *p* <.001, *d* = 0. 27 (Table 2).

**Table 2:** Behavioral data—Item Memory for the confidence responses

	Event boundary		No-event boundary	
	High-confidence	Low-confidence	High-confidence	Low-confidence
Pr score	0.36 (0.17)	0.14 (0.13)	0.32 (0.16)	0.15 (0.12)
Hit rate	0.41 (0.19)	0.26 (0.14)	0.36 (0.19)	0.27 (0.14)
FA rate	0.05 (0.05)	0.12 (0.08)	0.05 (0.05)	0.12 (0.08)

*Note*. Mean proportions of hits, false alarms, and Pr scores for each condition. Standard deviations are given in parentheses. *FA =* false alarm

To examine whether the likelihood of remembering one of the words preceding the critical word, given that the other preceding word was also remembered, was higher in the event boundary condition than in the no-event boundary condition, we conducted a mixed-effects model analysis. The model included fixed effects for event boundary, first/second word remembered, and the centered position of the first/second word, as well as random intercepts by participants and by test word nested within block list. The final model for the first preceding word included random slopes only for event boundary and second word remembered by participants, and second word remembered by test word nested within block list. For the analysis of the second preceding word, the model included random slopes only for first word remembered by participants and by test word nested within block list. The results for remembering the first preceding word when the second preceding word was remembered revealed no significant interaction between event boundary and memory performance for preceding word (*b* = 0.05, *SE* = 0.04, *z* = 1.7, *p* = 0.08). Similarly, the analysis for remembering the second preceding word when the first preceding word was remembered also showed no significant interaction (*b* = − 0.01, *SE* = 0.03, *z* = − 0.5, *p* =.6). While encountering an event boundary strengthened memory for the boundary word itself, the integration of different words from the preceding event remained unaffected.

### ERP results

#### Event-boundary N400 effect

**Fig. 4:**
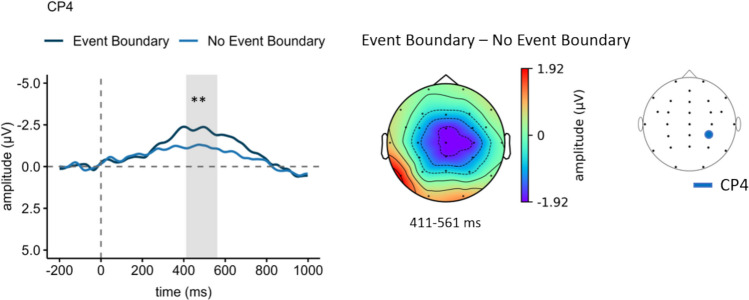
Grand average ERP responses at electrode CP4 depicting the N400 effect. The gray bar denotes the N400 time window (411–561 ms). The topographic map depicts the N400 effect (event boundary – no-event boundary) in the N400 time window

Mean amplitudes in the 411–561 ms time window were analyzed using two-way ANOVAs with factors AP (anterior–posterior) and event boundary (boundary, no-boundary). As apparent from Fig. 4, critical words in the event boundary condition elicited a larger N400 amplitude (*M* = − 1.9 µV, *SEM* = 0.08) compared with critical words in the no-event boundary condition (*M* = − 1.08 µV, *SEM* = − 0.08). A two-way ANOVA with factors event boundary and AP revealed a significant main effect of event boundary (*F*(1, 39) = 11.12, *p* =.002, *η*_*p*_^2^ =.22).

Thus, the analysis demonstrated a widely distributed N400 effect related to event boundaries, with ongoing events showing a diminished N400 amplitude compared with those that signaled the start of a new activity.

#### Subsequent memory effect (SME) for critical items

**Fig. 5:**
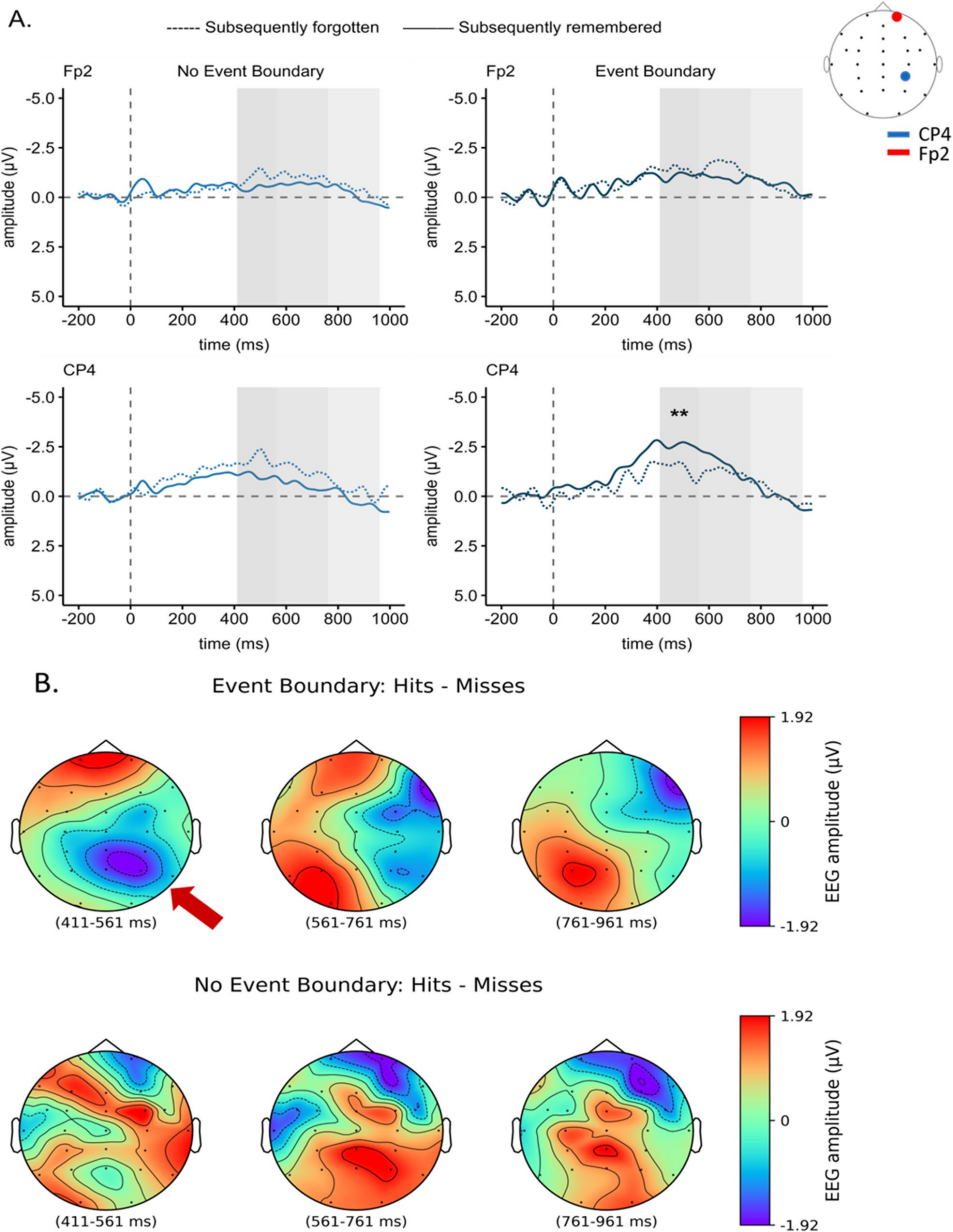
Subsequent memory effect (SME) for critical words. **A.** The ERP waveforms elicited at representative scalp electrodes by the onset of the critical words during the study phase. Shaded areas indicate the 411–561 ms, 561–761 ms, and 761–961 ms time windows in which mean amplitudes were analyzed. **B.** The topographic maps for the SME at the critical words within these time windows. The arrow indicates the time window in which hits elicited more negative ongoing waveforms than misses

Figure 5A illustrates the ERPs elicited by critical words that were subsequently remembered or forgotten. Mean amplitudes were analyzed separately for three consecutive time windows (411–561 ms, 561–761 ms, 761–961 ms) using three separate three-way ANOVAs, with factors of AP (anterior–posterior), event boundary (boundary, no-boundary), and memory (hits, misses).

##### N400 time window

The analysis of mean amplitudes in the N400 (411–561 ms) time window revealed a significant main effect of event boundary (*F*(1, 39) = 4.67, *p* =.04, *η*_*p*_^2^ = 0.11), an interaction between event boundary and memory (*F*(1, 39) = 5.31, *p* =.03, *η*_*p*_^2^ = 0.12), and an interaction between event boundary, AP and memory (*F*(1, 39) = 4.25, *p* =.05, *η*_*p*_^2^ = 0.10). To further explore the latter interaction, mean amplitudes were analyzed separately for anterior, and posterior electrode sites using ANOVAs with event boundary and memory as factors. At anterior sites, this follow-up analysis revealed a main effect of event boundary (*F*(1, 39) = 4.70, *p* =.04, *η*_*p*_^2^ = 0.11), however no significant interaction between factors. For posterior electrodes, the analysis revealed a significant interaction between event boundary and memory (*F*(1, 39) = 7.85, *p* =.008, *η*_*p*_^2^ = 0.12). To further investigate this interaction, subsequent memory effects (SME) were calculated as the difference between subsequently remembered (hits) and forgotten (misses) critical words, separately for the event boundary and no-event boundary conditions. A *t*-test revealed that in the event boundary condition, subsequently remembered critical words elicited a larger (i.e., more negative) N400 amplitude compared to subsequently forgotten ones, *t*(39) = − 2.65, *p* =.01, *d* = − 0.42 (hits – *M* = − 2.47, *SEM* = 0.29; misses – *M* = − 1.44, *SEM* = 0.32). In the no-event boundary condition, the difference between later-remembered and forgotten critical words did not reach statistical significance, *t*(39) = 1.62, *p* =.1, *d* = 0.26 (hits – *M* = − 1.05, *SEM* = 0.22; misses – *M* = − 1.85, *SEM* = 0.47).

##### 561–761 ms and 761–961 ms time windows

No significant main effects or interactions involving memory and/or event boundary were found as all *p*-values >.10.

In summary, in the two late time windows (561–761 ms vs. 761–961 ms), we neither found a difference between subsequently remembered and forgotten words across event boundary conditions nor a modulation of the SME by event boundary condition. However, a clear subsequent memory effect emerged in the N400 time window. This SME in the N400 interval took the form of more negative amplitudes for successfully remembered words as compared to forgotten words for critical words in the event boundary condition. This pattern was not observed in the no-boundary condition.

#### Retrograde subsequent memory effect (SME)

**Fig. 6:**
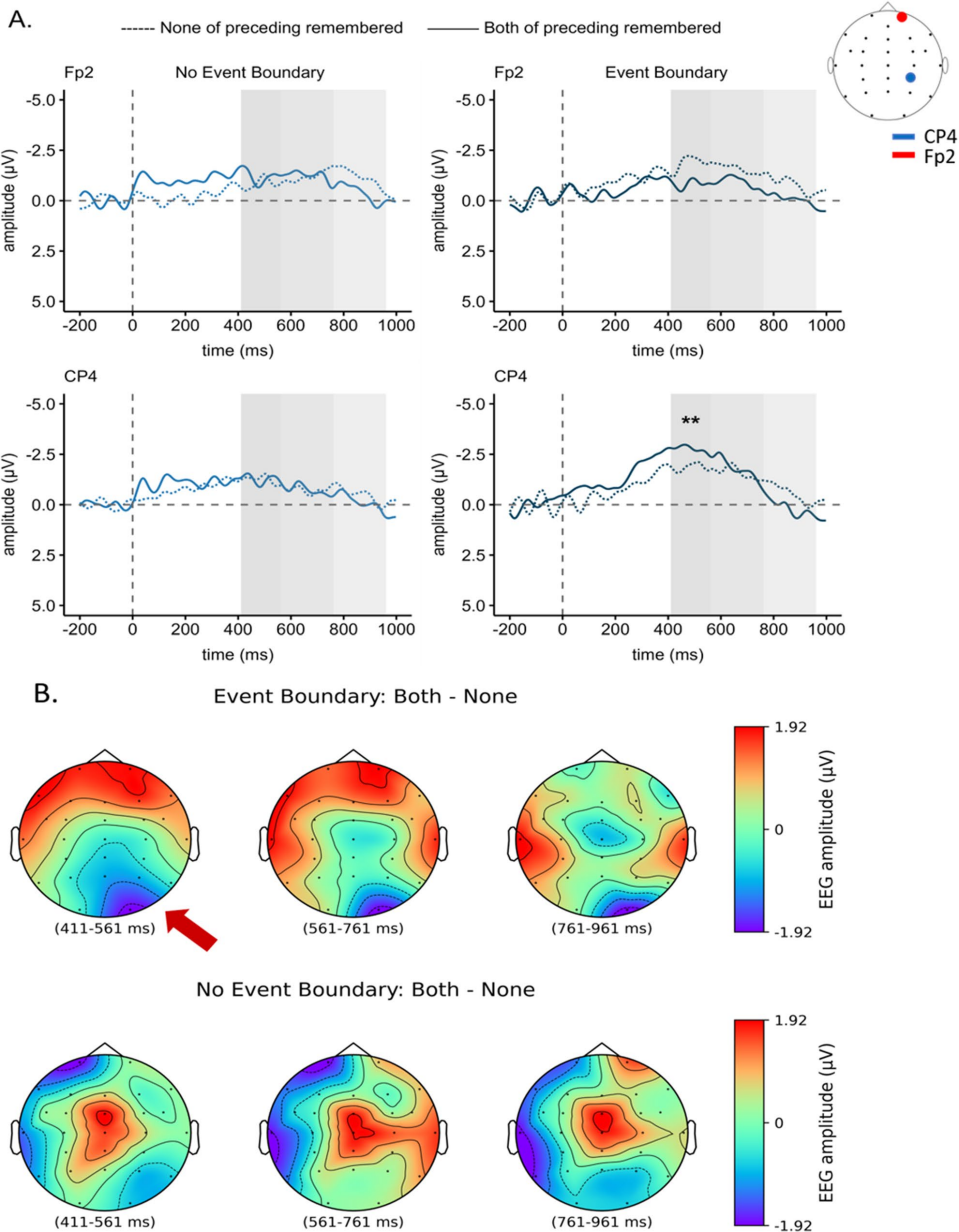
Retrograde subsequent memory effect (SME) for critical words. **A.** The ERP waveforms elicited at representative scalp electrodes by the onset of the critical words during the study phase. Shaded areas indicate the 411–561 ms, 561–761 ms, and 761–961 ms time windows in which mean amplitudes were analyzed. **B.** The topographic maps of the retrograde SME; arrow marks the time window in which trials with both preceding words remembered elicited more negative ongoing waveforms than trials with none remembered

 Due to the low number of trials involving critical words for which none of the preceding items were subsequently remembered, five participants had to be excluded from this analysis, resulting in a final sample of 35 participants for this part of the results. Figure 6A illustrates that the ERPs elicited by the critical words when both preceding words were later remembered and when neither was remembered. Mean amplitudes were analyzed separately for three consecutive time windows (411–561 ms, 561–761 ms, 761–961 ms) by using three-way ANOVAs, with factors AP (anterior–posterior), event boundary (boundary, no-boundary), and retrograde memory (both preceding words remembered vs. none remembered).

##### N400 time window

The analysis of mean amplitude in the 411–561 ms (N400) time window revealed a marginally significant effect of event boundary (*F*(1, 39) = 3.62, *p* =.06, *η*_*p*_^2^ = 0.10), and a significant interaction between event boundary, AP and memory (*F*(1, 34) = 11.26, *p* = 0.002, *η*_*p*_^2^ = 0.25). To further explore the interaction, mean amplitudes were analyzed separately for anterior, and posterior electrode sites using ANOVAs with event boundary and memory as factors. This follow-up analyses revealed no significant main effect or interaction at the anterior electrodes; however, at the posterior electrodes, analysis revealed a significant main effect of event boundary (*F*(1, 34) = 5.53, *p* =.025, *η*_*p*_^2^ = 0.14) and a significant interaction between factors (event boundary and memory) (*F*(1, 34) = 4.22, *p* =.05, *η*_*p*_^2^ = 0.11).

To further examine this significant interaction, retrograde SMEs were calculated separately for the event boundary and no-event boundary conditions. A *t*-test revealed that in the event boundary condition, critical words elicited a larger N400 amplitude when both preceding words were subsequently remembered, compared with when neither was remembered, *t*(34) = − 2.61, *p* =.01, *d* = − 0.44 (Both: *M* = − 2.63, *SEM* = 0.34; None: *M* = − 1.56, *SEM* = 0.32). In the no-event boundary condition, the SME at critical words did not reach statistical significance, *t*(34) = 0.37, *p* =.7, *d* = 0.06 (both: *M* = − 1.13, *SEM* = 0.31; none: *M* = − 1.33, *SEM* = 0.45).

##### 561–761 and 761–961 ms time windows

In the analysis of mean amplitudes in the last time windows (561–761 ms vs. 761–961 ms), no significant main effects or interaction involving memory and/or event boundary were found (all *p*-values >.1).

In summary, the findings of the retrograde SME demonstrate that in the early time window, critical words in the boundary condition elicited a greater N400 amplitude when both preceding words were later remembered compared with when neither was remembered. However, this retrograde SME was not observed in the no-boundary condition, where no significant differences emerged.

### Exploratory analysis

#### Topographical profile analyses

To determine whether the topographic profiles of the N400 effect and the subsequent memory effects (SMEs) differ qualitatively rather than just in relative strength (Urbach & Kutas, 2002, 2006), we applied vector scaling following McCarthy and Wood (1985) and conducted effect × AP × laterality ANOVAs for the relevant N400 and subsequent memory effects. We report only significant effects that involve the effect factors. Our analysis focused on three key topographical contrasts, all within the N400 time window: (1) the comparison between the N400 effect and the SME for critical words, (2) the comparison between the N400 effect and the retrograde SME, and (3) the comparison between the SME for critical words and the retrograde SME.

To calculate the N400 effect, we computed the mean amplitude difference between no-event boundary and event boundary trials for each participant in the N400 time window. Subsequent memory effects were calculated by subtracting the mean amplitudes of forgotten trials from those of remembered trials, as well as subtracting the mean amplitudes of trials where neither preceding word was remembered from those where both were remembered. Finally, all scores were vector-scaled.

#####  1 st contrast: N400 vs. SME for critical words

The first contrast examined whether the spatial distribution of the N400 effect was comparable to that of the SME for critical words. Given their similar temporal characteristics, such a finding would support the assumption that the N400-SME is functionally related to the N400 effect. An ANOVA with the factors effect (N400, SME for critical words in the event boundary condition), AP (frontal, frontocentral, central, centroparietal, parietal), and laterality (left, middle, right) revealed a significant interaction between effect and AP, *F*(4, 156) = 4.59, *p* =.02, *η*_*p*_^2^ = 0.11. These results indicate that the N400 and the SME for critical words differ qualitatively in that the N400 has a more central distribution, whereas the SME for critical words is most pronounced over the right parietal region (Figs. 4 and 5B).

##### 2nd contrast: N400 vs. Retrograde SME

Next, we investigated whether the N400 effect and the retrograde SME differed in their topographic profiles. In the N400 (411–561 ms) time window, an ANOVA with the factors effect (N400, retrograde SME in the event boundary condition), AP, and laterality revealed a significant interaction between effect and AP, *F*(4, 136) = 10.19, *p* <.001, *η*_*p*_^2^ = 0.23, indicating that the retrograde SME has a distinct topographic profile compared with the N400 effect (Figs. 4 and 6B).

##### 3rd contrast: SME for critical words vs. Retrograde SME

Lastly, we examined whether the SME for critical words and the retrograde SME differed in topographic distribution in the N400 time window (411–561 ms). An ANOVA with the factors effect (SME for critical words vs. retrograde SME, both in the event boundary condition), AP, and laterality revealed a significant effect × AP interaction (411–561 ms), *F*(4, 136) = 3.76, *p* =.04, *η*_*p*_ = 0.10, suggesting that within the N400 time window, the SME for critical words, and the retrograde SME exhibited distinct topographic distributions. The SME for critical words shows a centroparietal distribution whereas the retrograde SME is predominantly right-parietal (Figs. 5B and 6B).

To summarize, the results indicate that the N400 has a distinct topographic profile from both the SME for critical words and the retrograde SME. Specifically, while the N400 is more centrally distributed, the SME for critical words and the retrograde SME are primarily centroparietal and right parietal, respectively. Furthermore, the SME for critical words and the retrograde SME exhibited different topographic distributions.

## Discussion

Event boundaries have been shown to trigger memory updating processes and exert a lasting impact on how events are remembered. Nevertheless, their role in modulating neural predictive processing and memory is still not well understood. In the present study, we used ERP subsequent memory effects elicited by critical words embedded in spoken narratives to explore how event boundary processing interacts with memory encoding, focusing on how predictive processing shapes memory formation and retrieval in naturalistic contexts.

### Behavioral results

A substantial body of research highlights the role of event boundaries in memory encoding. Studies by Heusser et al. (2018) and Swallow et al. (2009) found that objects presented at event boundaries were better remembered than those presented within continuous events. Consistent with these findings, the present study demonstrated better memory for critical words indicating an event boundary compared with critical words marking no event boundary, supporting the notion that event boundaries act as “cognitive anchor points” for memory encoding (DuBrow et al., 2024). This view aligns with event segmentation theory (EST), which posits that event boundaries are critical points where active memory for the current event is reset and updated. Objects present at boundaries receive enhanced processing, including relational and associative encoding within the context of the model of the upcoming event, leading to more readily accessible long-term representations (Swallow et al., 2011; Zacks et al., 2007). This enhancement likely reflects not a single mechanism but the interplay of several processes critical for event model updating, including enhanced sequential order encoding as indicated by the coupling of theta and gamma EEG oscillations (Heusser et al., 2016) or memory reactivation triggered by the detection of mismatch with prior context (Silva et al., 2019). Additionally, the enhanced memory for boundary words is also consistent with the growing body of literature emphasizing the critical role of contextual stability in organizing episodic memory (Clewett et al., 2019; DuBrow & Davachi, 2016). As in our stimulus design, event boundaries represented a transition between two distinct periods of narrative stability, marked by the abrupt change in activity or goal structure. The enhanced memory for boundary words is thus explained by the cognitive system prioritizing the encoding of information that serves as entry point or anchor for the subsequent stable event model (Clewett et al., 2019).

Furthermore, our confidence-based analysis showed that the memory advantage for boundary words was more pronounced for high as compared to low confidence responses. This pattern suggests that the memory advantage for the critical words indicating a boundary is particularly pronounced for trials associated with stronger memory traces (Swallow et al., 2009). High-confidence responses typically reflect more robust or accessible memory representations (Yonelinas, 2002), indicating that event boundaries promote deeper encoding of the boundary-relevant information (Heusser et al., 2018; Zacks et al., 2007). Despite clear evidence that boundary words were better remembered, we did not observe a memory benefit for a preceding word when the other preceding word was successfully recalled. Based on theories suggesting that prediction errors at boundaries initiate storage of prior event traces (Zacks et al., 2007), which can include a rapid memory replay of the just-encoded event to promote its successful encoding, we hypothesized that this process would enhance the associative binding between the two preceding words in the event boundary condition. Consequently, we expected a higher likelihood of remembering one preceding word if the other was remembered in the event boundary condition, reflecting this strengthening of preceding events. However, conditionalized item memory did not differ between event boundary and no-event boundary conditions. One explanation is that the end of a narrative may have acted as a subtle “fine boundary” (Zwaan & Radvansky, 1998), enhancing memory even in the no-boundary condition. Alternatively, semantic congruency between preceding words and the critical word in the no-event boundary condition may have also strengthened memory traces, reducing the difference between conditions (Höltje & Mecklinger, 2022).

### Event-boundary N400 effect

We also examined how event boundaries in narrative discourse are processed online by investigating how boundary predictability is reflected in ERPs. In our stimulus design, boundaries were indicated through an unexpected goal shift (e.g., “shopping” after “borrowing books”), creating the type of informational discontinuity that EST describes as a prediction error (Kurby & Zacks, 2008). The results indicated an N400 effect for event boundaries, with boundaries eliciting larger (more negative) N400 amplitudes than the no-event boundary condition, reflecting the increased conceptual-semantic processing demands triggered by the prediction error. This finding aligns with previous ERP research on event boundaries (Delogu et al., 2018) and is consistent with a substantial body of work on semantic sentence violations (DeLong et al., 2005; Hubbard & Federmeier, 2024; Kutas & Federmeier, 2011; Kutas & Hillyard, 1980). The N400 is sensitive to how well a word can be integrated in its preceding context (Kutas & Federmeier, 2011), with larger (more negative) N400 amplitudes for words that are less predictable or more difficult to integrate.

An alternative, though related explanation for the event-boundary N400 effect observed here comes from the structure building framework (SBF) (Gernsbacher, 1990). According to SBF, a “shift” is initiated when incoming information is either inconsistent with, or irrelevant to, the current active mental structure, causing mapping failure (Gernsbacher, 1990; Zacks et al., 2009). This shift mechanism in SBF is considered functionally similar to the segmentation process in EST (Zacks et al., 2009). Thus, the N400 observed at boundary words might reflect the difficulty integrating the current, unpredicted word with prior narrative information, signaling the cognitive disruption caused by this mapping failure (Chwilla & Kolk, 2005; Delogu et al., 2018; Gernsbacher, 1990; Zacks et al., 2009).

Thus, words at event transitions require greater cognitive effort for comprehension and integration into the evolving mental representation of the situation (Bailey & Zacks, 2015). When a context shift occurs, individuals face increased demands in retrieving and integrating conceptual knowledge that fits the new situation. The larger N400 could reflect this heightened effort to access and process relevant semantic information pertinent to the new context initiated by the boundary (Chwilla & Kolk, 2005; Lau et al., 2016).

### Subsequent memory effect for critical words

In the present study, ERP results indicated that in the event boundary condition, subsequently remembered critical words elicited a larger (more negative) N400 amplitude than forgotten ones. This negative-going SME contrasts with typical findings from single-word recognition tasks, where remembered items usually elicit more positive ERPs. Our results suggest that at event boundaries, successful memory for critical words may be associated with increased conceptual-semantic processing demands required to resolve the contextual shift (as reflected by a larger N400) (Delogu et al., 2018; Höltje & Mecklinger, 2022) or with a greater allocation of resources to processing these words (Clewett et al., 2019; DuBrow et al., 2024). In contrast, the no-boundary condition showed a non-significant trend toward more positive amplitudes in the N400 time-window for remembered words.

The EST proposes that event boundaries arise when expectations about an ongoing situation are disrupted, resulting in a prediction error (Kurby & Zacks, 2008; Zacks et al., 2007). A negative SME at boundaries could reflect the neural processes involved in detecting and responding to this prediction error, particularly if successfully processing the error enhances memory for subsequent information (i.e., the critical word) (Schlichting & Preston, 2015). Thus, the larger negative amplitude for remembered critical words reflects the mobilization of resources needed to integrate unpredicted critical words and make sense of the context shift, thereby forming a durable memory for these words (Greve et al., 2017; Henson & Gagnepain, 2010; Höltje & Mecklinger, 2022). According to Tulving et al. (1994), the retrieval of information from semantic memory often supports episodic memory encoding, especially for novel information. Hence, the processing of prediction errors at event boundaries necessitates conceptual-semantic integration, which in turn supports the encoding of new episodic information into episodic memory. As the system attempts to resolve the context shift, this increased processing may help integrate the unexpected word into a new, stable event (Heusser et al., 2018; Swallow et al., 2009). Therefore, the N400-SME, while triggered by prediction error, highlights the moment by moment neural effort dedicated to re-establishing a coherent and stable mental representation of the ongoing narrative, which ultimately leads to a durable memory trace for the new event (Clewett et al., 2019; Güler et al., 2025).

Additionally, topographic analysis revealed that the N400, linked to conceptual-semantic retrieval and centrally distributed, differs topographically from the SME for critical words, with its more centroparietal to right parietal distribution. This suggests that, although they overlap in time, these effects arise from partially distinct interplays of neural activity. In turn, this may indicate that, beyond N400-related conceptual-semantic processing demands, additional processes such as attention or cognitive effort also contributed to successful encoding, as reflected in the N400 SME.

### Retrograde subsequent memory effect

Furthermore, this study aimed to investigate how event boundaries influence memory for preceding information and to characterize the neural mechanisms underlying the retrograde subsequent memory effect (SME). Retrograde strengthening of memory has primarily been observed for events with emotional significance, as demonstrated by retrograde memory enhancement (RME), where emotional arousal experienced after a neutral event boosts the consolidation of recently experienced information (Anderson et al., 2006; Patil et al., 2017). Our results indicated that the critical words elicited a larger negative^3^ amplitude in the N400 time window when both of the preceding words were subsequently remembered compared with when neither of the preceding words was remembered. This suggests that memory formation processes can operate retroactively when an event boundary occurs. One possible explanation is that neural activity at boundaries reflects rapid reinstatement of just-encoded information (Griffiths & Fuentemilla, 2020). Supporting this interpretation, a recent study showed that contrastive focus accent in spoken language can trigger the reinstatement of preceding sentence context, as reflected by a retrograde SME (Spalek et al., 2025). By reactivating recently encoded information, the brain may be strengthening its consolidation and by this enhancing its accessibility for later retrieval (Silva et al., 2019; Sols et al., 2017). Moreover, this post-encoding memory reinstatement is selective for learning meaningful episodic content that depicts coherent relations between elements but not for sequences of unrelated items (Wu et al., 2022). Thus, the retrograde SME might be interpreted as reflecting reinstatement-related processes, with event boundaries functioning as brief periods during which the brain consolidates recently experienced information. The negative polarity of the retrograde SME in the N400 time window is an interesting aspect. The larger N400 for both-remembered preceding words at boundaries may reflect deeper conceptual integration (Kutas & Federmeier, 2000) or the brain’s effort to reactivate preceding semantic information upon boundary detection. Alternatively, the increased negativity at boundaries when both preceding words were remembered may reflect a mismatch or prediction error signal (Silva et al., 2019), which in turn triggers a stronger reactivation of preceding information as the brain attempts to resolve the discrepancy. Such reactivation may facilitate the integration of earlier content with incoming information in the unfolding narrative, promoting the formation of a cohesive memory representation (Silva et al., 2019; Sols et al., 2017). It is worth noting that the scalp distribution (right parietal) of the observed effect differs from that reported by Silva et al. (2019) who observed a left-lateralized anterior distribution. Whereas Silva and colleagues used movie clips to induce boundaries, our study employed naturalistic narratives in which boundaries were defined by shifts in described actions or goals, and different post-boundary onsets were analyzed. These methodological differences, including stimulus modality (narrative text vs. movie clips), analyzed time window (411–561 ms vs. 600–1400 ms), and methodological approaches (ERP amplitude vs. pattern similarity) likely account for the qualitative differences in topographical patterns. Our findings highlight that memory formation in naturalistic contexts is an active process shaped by event segmentation, predictive processing, and consolidation. Uniquely, we combined ecologically valid auditory stories with clearly manipulated event boundaries with an ERP subsequent memory (SME) approach, an experimental design not applied previously in this domain. Our study provides novel electrophysiological evidence that detecting a clearly defined context shift (or event boundary) when listening to naturalistically spoken narratives both demands semantic integration of the new event and may trigger retroactive reinstatement of the preceding context. Taken together, these results offer valuable insights into how the brain transforms ongoing continuous experience into episodic memories for real life events and provides compelling evidence that successful encoding of event boundaries relies on the activation of conceptual semantic knowledge, as indexed by the N400 component.

## Data Availability

The statement regarding data availability is included under “Open Practice Statements”.

## Footnotes

1 This deviation from the preregistered exclusion criteria was made to avoid replacing participants.

2 Note that confidence-based analyses for ERP data are not reported in the *Results* section owing to limited number of trials.

3 Note that we expected a positive instead of a negative polarity retrograde SME as at the time of preregistration, we were not aware of the study by Silva et al. (2018) that reported negative retrograde SME.

## Declarations

### Open practices statements

The stimulus materials for the experiments are available upon request and the experiment was preregistered. Behavioral and EEG data and analysis scripts to reproduce the results are available at https://osf.io/f624h/?view_only=653066e2959f46c7872a059713d55563
